# Risk factors associated with physician trainee concern over missed educational opportunities during the COVID-19 pandemic

**DOI:** 10.1186/s12909-021-02665-0

**Published:** 2021-04-17

**Authors:** Sunny S. Lou, Charles W. Goss, Bradley A. Evanoff, Jennifer G. Duncan, Thomas Kannampallil

**Affiliations:** 1 Department of Anesthesiology, Washington University School of Medicine, 660 S. Euclid Avenue, Campus Box 8054, St Louis, MO 63110 USA; 2 Division of Biostatistics, Washington University School of Medicine, St Louis, MO USA; 3 Department of Medicine, Washington University School of Medicine, St Louis, MO USA; 4 Department of Pediatrics, Washington University School of Medicine, St Louis, MO USA; 5 Institute for Informatics, Washington University School of Medicine, St Louis, MO USA

**Keywords:** COVID-19, Residency, Graduate medical education, Surgical training, Missed clinical opportunities, Physician burnout

## Abstract

**Background:**

The COVID-19 pandemic resulted in a transformation of clinical care practices to protect both patients and providers. These changes led to a decrease in patient volume, impacting physician trainee education due to lost clinical and didactic opportunities. We measured the prevalence of trainee concern over missed educational opportunities and investigated the risk factors leading to such concerns.

**Methods:**

All residents and fellows at a large academic medical center were invited to participate in a web-based survey in May of 2020. Participants responded to questions regarding demographic characteristics, specialty, primary assigned responsibility during the previous 2 weeks (clinical, education, or research), perceived concern over missed educational opportunities, and burnout. Multivariable logistic regression was used to assess the relationship between missed educational opportunities and the measured variables.

**Results:**

22% (301 of 1375) of the trainees completed the survey. 47% of the participants were concerned about missed educational opportunities. Trainees assigned to education at home had 2.85 [95%CI 1.33–6.45] greater odds of being concerned over missed educational opportunities as compared with trainees performing clinical work. Trainees performing research were not similarly affected [aOR = 0.96, 95%CI (0.47–1.93)]. Trainees in pathology or radiology had 2.51 [95%CI 1.16–5.68] greater odds of concern for missed educational opportunities as compared with medicine. Trainees with greater concern over missed opportunities were more likely to be experiencing burnout (*p* = 0.038).

**Conclusions:**

Trainees in radiology or pathology and those assigned to education at home were more likely to be concerned about their missed educational opportunities. Residency programs should consider providing trainees with research or at home clinical opportunities as an alternative to self-study should future need for reduced clinical hours arise.

**Supplementary Information:**

The online version contains supplementary material available at 10.1186/s12909-021-02665-0.

## Background

The novel coronavirus disease 2019 (COVID-19) emerged in December of 2019 [1] and spread around the world, thus far leading to over a million deaths [2]. In the face of limited resources and the inherent uncertainty of the pandemic, healthcare systems were forced to rapidly adapt, restructuring patient admissions and implementing social distancing measures to increase capacity and reduce risk of disease transmission for both patients and healthcare providers [3–6].

These changes had considerable impact on all aspects of medical practice, including residency and fellowship training programs [7–11]. During the early stages of the pandemic, there was a reduction in elective surgical cases [3, 12], and a decrease in non-COVID related patient healthcare utilization even for urgent conditions [13–16]. This led to a reduction in usual clinical volume for both medical and surgical specialties [17–19], decreasing clinical training opportunities. In pandemic hot spots, trainees may have been re-assigned outside of their specialty to provide COVID-19 care. Otherwise, with low patient census and to minimize COVID-19 exposure risk, many institutions reduced trainees’ clinical time and required them to work on education or research at home [9, 10, 20]. Furthermore, social distancing guidelines resulted in the cancellation of in-person didactics, necessitating a reconfiguration of resident education to online or virtual platforms [21–23], limiting formal didactic opportunities for trainees. These changes potentially have consequences not only for trainee preparedness for independent practice, but also for their personal well-being [10].

We investigated the effect of the COVID-19 pandemic on trainee educational opportunities to address the following research questions: (1) what is the prevalence of trainee concern for missed educational opportunities; (2) what are the risk factors leading to such concerns; and (3) is there a relationship between concern for missed educational opportunities and wellness.

## Methods

### Participants and survey

All physician trainees (*n* = 1375) at the Washington University School of Medicine, Barnes-Jewish Hospital, and St. Louis Children’s Hospital were invited to participate in a voluntary de-identified web-based survey on May 13, 2020. A follow-up reminder was sent 1 week later. A $50 gift card raffle was offered to survey respondents for participation. This survey was part of a longitudinal study assessing the wellness of physician trainees during the COVID-19 pandemic [24].

During this period at our institution, surgical volume was approximately 33% lower and inpatient admissions were approximately 20% lower than pre-pandemic levels. The nadir of patient volume was 1 month prior, when the volume of surgeries decreased by nearly 65% and inpatient admissions were down by 40%.

The survey included questions from several domains. Demographic information was collected relating to gender, ethnicity, marital status, and parenthood status. Clinical responsibilities were assessed based on clinical role (resident, fellow), clinical specialty, and the type of assigned primary responsibility over the previous 2 weeks (i.e., clinical–inpatient, clinical–outpatient, education–at home, research–at home, research–on campus). Exposure to patients testing positive for COVID-19 was also assessed.

This study was approved by the institutional review board of Washington University. Prior to completing the survey, all participants read an information sheet that included details of the study; by completing the survey, participants provided consent to participate in this research study (IRB #202004021, Washington University).

### Outcomes

The primary outcome was the impact of COVID-19 on trainee education, assessed with the prompt “How stressed are you about missed educational opportunities” on a 5-point scale ranging from “not at all” to “extremely.”

Secondary outcomes related to burnout and professional fulfillment were assessed using the Stanford Professional Fulfillment Index (PFI) [25]. For a full list of survey questions, please see the Additional file 1.

### Statistical analysis

Gender was categorized as Female/Not Female. Ethnicity was categorized as Caucasian/Non-Caucasian. Marital Status was categorized as Married/Not Married.

Clinical specialty was categorized into three groups: surgery, reduced patient contact, and medicine. Surgery included all specialties that required significant operating room time and thus would have been most affected by the decrease in surgical volume: general surgery, neurosurgery, orthopedic surgery, obstetrics and gynecology, ophthalmology, otolaryngology, plastic surgery, urology, and anesthesiology. Reduced patient contact included two specialties with reduced direct patient contact: pathology and radiology. Medicine included the remainder of the specialties: internal medicine, pediatrics, emergency medicine, neurology, child neurology, psychiatry, dermatology, physical medicine and rehabilitation, radiation oncology, and medical genetics.

Primary responsibility was categorized into three groups: clinical, education and research. Clinical included all respondents reporting spending the majority of their time doing clinical work (i.e., inpatient, outpatient, or clinical work from home); education included all respondents reporting the majority of their time spent on “education–at home”; research included all respondents reporting the majority of their time conducting research either at home or on campus.

The primary outcome, concern for missed educational opportunities, was analyzed as a dichotomous outcome variable with responses of “somewhat,” “quite a bit,” or “extremely,” categorized as “concerned,” and “not at all” or “a little” categorized as “not concerned.”

The secondary outcome, burnout, was determined from an average item score for the workload and depersonalization scales (score range 0 to 4), using a cut-point of 1.33 [25], where scores greater than or equal to 1.33 was considered as “burned out.” Similarly, for professional fulfillment, an item score of greater than or equal to 3.0 was used as the cut-point (scale range 0–4), which has been shown to correlate with physicians indicating their quality of life as being “very good.” [25]

For univariable analyses of the association between demographic and clinical variables with the missed educational opportunity outcome, chi-square tests were used. Variables with *p* < 0.10 in univariable testing were incorporated in a multivariable binary logistic regression model. Variables with p < 0.10 in the multivariable model were retained and further investigated with pairwise comparisons. Wald test was used for all comparisons within the multivariable model. All analyses were conducted in R 3.6.1 [26].

## Results

### General characteristics

There were 301 responses to the survey for a response rate of 21.8% (301/1375). Participants were predominantly residents (66%), female (56%), Caucasian (64%), married (55%), and without children (78%). 25% reported exposure to a COVID-19 patient.

Specialties with a strong operating room focus (i.e., surgery and anesthesiology) comprised 26% of the participants, while reduced direct patient contact specialties (i.e., pathology, radiology) comprised 12%, with the remainder of the participants categorized as medicine (62%). 73% of respondents reported spending the majority of their time over the previous 2 weeks doing clinical work, while 13% reported doing education at home, and 15% were performing research. Overall, 47% (142/301) of the participants were concerned about missed educational opportunities.

### Univariable analysis

In the univariable analysis (see Table 1), differences in parenthood status (*p* = 0.028), clinical specialty (*p* = 0.018), and assigned primary responsibility (*p* = 0.020) were associated with the concern for missed educational opportunities primary outcome.

**Table 1:** Univariable comparison between demographic factors, COVID+ patient exposure, specialty, and clinical responsibilities with concern over missed clinical opportunities. Responses were grouped into Not concerned (Not at all, a little) and Concerned (somewhat, quite a bit, extremely)

			***Currently how stressed are you about missed clinical opportunities?***		
Variable	Group	Total	Not concerned	Concerned	*P*-value
Clinical Role	Fellow	101 / 301 (33.6%)	50 / 101 (49.5%)	51 / 101 (50.5%)	0.485
	Resident	200 / 301 (66.4%)	109 /200 (54.5%)	91 / 200 (45.5%)	
Female	Yes	168 / 301 (55.8%)	87 / 168 (51.8%)	81 / 168 (48.2%)	0.273
	No	133 / 301 (44.2%)	72 / 133 (54.1%)	61 / 133 (45.9%)	
Caucasian	Yes	192 / 301 (63.8%)	98 / 192 (51.0%)	94 / 192 (49.0%)	0.483
	No	109 / 301 (36.2%)	61 / 109 (56.0%)	48 / 109 (44.0%)	
Married	Yes	167 / 301 (55.5%)	82 / 167 (49.1%)	85 / 167 (50.9%)	0.184
	No	134 / 301 (44.5%)	77 / 134 (57.4%)	57 / 134 (42.5%)	
Children	Yes	67 / 301 (22.3%)	27 / 67 (40.3%)	40 / 67 (59.7%)	0.028 *
	No	234 / 301 (77.7%)	132 / 234 (56.4%)	102 / 234 (43.6%)	
COVID+ exposure	Yes	76 / 301 (25.2%)	38 / 76 (50.0%)	38 / 76 (50.0%)	0.662
	No	225 / 301 (74.8%)	121 / 255 (53.8%)	104 / 255 (46.2%)	
Specialty	Medicine	182 / 294 (61.9%)	107 / 182 (55.7%)	75 / 182 (44.3%)	0.018 *
	Surgery	76 / 294 (25.9%)	39 / 76 (51.3%)	37 / 76 (48.7%)	
	Radiology Pathology	36 / 294 (12.2%)	12 / 36 (33.3%)	24 / 36 (66.7%)	
Primary responsibility	Clinical	204 / 281 (72.6%)	111 / 204 (54.4%)	93 / 204 (45.6%)	0.020 *
	Education	36 / 281 (12.8%)	11 / 36 (30.6%)	25 / 36 (69.4%)	
	Research	41 / 281 (14.6%)	24 / 41 (58.5%)	17 / 41 (41.5%)	

60% of trainees with children had concern for missed opportunities as compared with 44% of those without children. 67% of radiology or pathology trainees were concerned about missed opportunities as compared with 49% of trainees in surgical specialties and 44% of medicine trainees. Finally, 69% of trainees primarily assigned to education at home were concerned about missed opportunities as compared to 41% of trainees conducting research and 45% of trainees performing clinical work.

### Multivariable analysis

Variables with *p* < 0.10 in the univariable analyses were included in the multivariable binary logistic regression model (Table 2). Within the multivariable model, all variables with p < 0.10 were retained and explored further with pairwise comparisons where applicable. All three variables met this cutoff (children at home *p* = 0.061, primary responsibility *p* = 0.029, specialty group *p* = 0.068) and were retained in the final model. Adjusted odds ratios are reported in comparison to the reference group of trainees without children assigned primarily to clinical work in a medicine specialty.

**Table 2:** Multivariable binary logistic regression for predictors of concern for missed clinical opportunities. The reference group is physicians without children primarily assigned to clinical duties in a medicine (i.e., non-surgery / radiology / pathology) specialty

Variable	Group	Adjusted odds ratio	95% confidence interval	*P*-value
Children	Yes	1.75	0.98–3.17	0.061
Specialty	Medicine	1.00	Reference group	
	Surgery	1.28	0.72–2.27	0.395
	Radiology/ Pathology	2.52	1.16–5.68	0.021 *
Primary responsibility	Clinical	1.00	Reference group	
	Education	2.85	1.32–6.45	0.009 *
	Research	0.96	0.47–1.93	0.913

After adjusting for covariates, trainees assigned to primarily engage in education at home had 2.85 [95% confidence interval (CI) of the adjusted odds ratio 1.33–6.45] greater odds of being concerned over missed educational opportunities as compared to trainees engaged in clinical work. Trainees performing research, whether at home or on campus, were not significantly more likely to be concerned in comparison to their clinical peers [adjusted odds ratio 0.96, 95% CI (0.47–1.93)].

Furthermore, trainees in pathology and radiology had over 2.5 [adjusted odds ratio 2.51, 95% CI 1.16–5.68] greater odds of concern over missed educational opportunities as compared to their medicine peers; meanwhile, surgical trainees were not significantly different from their medicine peers [adjusted odds ratio 1.28, 95% CI 0.72–2.27] nor from radiology / pathology trainees [adjusted odds ratio 0.51, 95% CI 0.21–1.18].

After adjusting for specialty and primary responsibility, the relationship between parenthood status and concern for missed opportunities was marginal [adjusted odds ratio 1.75, 95% CI 0.98–3.17, *p* = 0.061].

### Missed educational opportunities and burnout

In a secondary analysis for the relationship between concern for missed educational opportunities and burnout (Table 3), we found that 52% of trainees who reported being burned out were concerned about missed educational opportunities as compared with 42% in the non-burned out group (*p* = 0.035). Professional fulfillment was not significantly associated with concern for missed educational opportunities (*p* = 0.206).

**Table 3:** Association between concern for missed clinical opportunities and physician wellness; Not concerned (Not at all, a little), Concerned (somewhat, quite a bit, extremely)

			***Currently, how stressed are you about missed clinical opportunities?***		
Variable	Group	**Total**	Not concerned	Concerned	*P*-value
Burnout	Yes	111 / 296 (37.5%)	50 / 111 (45.0%)	61 / 111 (55.0%)	0.035 *
	No	185 / 296 (62.5%)	108 / 185 (58.4%)	77 / 185 (41.6%)	
Professional Fulfillment	Yes	70 / 296 (23.6%)	42 / 70 (60.0%)	28 / 70 (40.0%)	0.206
	No	226 / 296 (76.4%)	114 / 226 (50.4%)	112 / 226 (49.6%)	

## Discussion

In a cross-sectional survey of residents and fellows at a large academic medical center during the early part of the pandemic, we found that nearly half of trainees were concerned about missed educational opportunities as a result of the COVID-19 pandemic. Trainees more likely to be concerned were those primarily assigned to education at home and those specializing in radiology or pathology.

Due to overall decreased clinical volume and workload during the COVID-19 pandemic across the United States, an unprecedented percentage of trainees were asked to study at home or work on research projects rather than engage in clinical work. These decisions were based on a number of factors including protecting trainees from potential COVID-19 exposure, scarcity of personal protective equipment resources, and a reduction in clinical services [9, 20, 27]. We found that trainees assigned to spend the majority of their time studying at home had nearly three-fold greater odds of being concerned about missed opportunities compared to their peers assigned to clinical work (Table 2). Surprisingly, trainees who were able to spend their non-clinical time performing research were protected from such concerns. Furthermore, those that were stressed about missed opportunities were more likely to be burned out (Table 3), highlighting the mental health consequences of these shifts in trainee work responsibilities.

We speculate that trainees who used their non-clinical time for research maintained a perception that their actions were contributing to their careers and furthering their education in a way that being instructed to study at home was not. This highlights the potential need for residency and fellowship programs to consider incorporating structured research opportunities to maintain trainee engagement should the need arise again for reduced clinical time. Alternatively, perhaps identifying strategies to enable trainees to engage in clinical work at home would lessen the perception of missed educational opportunities during reductions in clinical volume. This can include participation in virtual consults, telehealth appointments, virtual rounds, or case conferences. For example, use of telehealth has rapidly expanded since the start of the COVID-19 pandemic [4], and trainee participation in telehealth has been strongly supported by the Accreditation Council for Graduate Medical Education [28]. Participation in telehealth represents new opportunities for trainee education [29] that likely will also prove useful for trainee future independent practice as telehealth opportunities continue to grow [30].

Much of the prior research on the impact of COVID-19 on graduate medical education has focused on the impact on surgical trainees due to the reduction in operative case volume [10, 31–40]. At our institution, the nadir of operative volume was in early April 2020 when surgical cases were approximately one third of pre-pandemic levels, similar to what has been reported elsewhere [32, 33]. Common findings across these studies include decreased trainee operative opportunities, altered rotation schedules, decreased work hours, and increased concern for the ability to meet required operative case minimums for graduation. However, previous research has not investigated whether surgical trainees are uniquely affected in comparison with their peers in other specialties, as outpatient visits and hospital admissions were also decreased during the same time period [17, 18]. Our data suggests that, surprisingly, pathology and radiology trainees had the highest odds of being concerned about missed educational opportunities, while surgical trainees were not much different from their medicine peers. The reasons for a higher prevalence of perceived lost educational opportunities among radiology and pathology trainees are likely multifactorial; not only did radiologists and pathologists see widespread reduction in overall clinical volume and work hours [41, 42], but also these specialties are best positioned for remote work. Many departments decided to limit resident-attending interactions to telephone or other virtual platforms to reduce disease transmission risk, perhaps unintentionally reducing the frequency and quality of these important educational interactions [43, 44]. Based on our data and others, there is a need for increased awareness of the challenges that radiology and pathology trainees are facing.

This study has several limitations. First, it was performed at a single academic medical center near the end of the first wave of infections (May 2020). Generalizability may be limited as other hospital systems may have experienced a lesser or greater reduction in clinical volume and likely made different choices in how training programs adapted in response. In addition, trainee sentiment may have been different at different points in the pandemic curve. However, the concerns regarding missed educational opportunities existed in other academic institutions [10, 31–41, 45, 46], and the reasons are likely similar. Second, the overall response rate was 22%; we cannot assume that respondents are a random sample of the total trainee population at our institution and thus results may not be representative. For example, trainees with fewer clinical responsibilities may have had more time to participate thus biasing our results. However, our survey response rate is comparable with other survey-based studies conducted on trainee physicians [10, 31, 47]. Third, due to the way we assessed primary work responsibility, we were unable to determine the relative proportion of time or number of hours spent performing clinical, educational or research tasks; trainees reporting they were primarily engaged in clinical work may have still spent some portion of their time on research or education and vice versa. Fourth, some of the comparisons that were performed (e.g., between specialty groups), were not sufficiently powered due to our response rate. Our assessment of missed educational opportunities was based on the self-reports of trainees and may have evolved during the course of the pandemic. However, these concerns, albeit during a period of heightened restrictions, highlight the need for academic institutions to develop strategies to engage trainees during periods of uncertainty. Further research is needed in evaluating the diversity of education at home experiences to identify if specific strategies were more effective than others, or if a different division of clinical versus educational time would have led to greater trainee satisfaction.

## Conclusions

Concerns about lost educational opportunities were widespread across all training programs during the early portion of the COVID-19 pandemic. Trainees in radiology or pathology and those assigned to education at home were more likely to be concerned as compared to their peers. While there may be no way to replace the lost educational opportunities, programs can support their trainees’ career development in other ways such as by providing research opportunities or opportunities to engage in clinical work from home during times of insufficient clinical volume.

## Supplementary Information

**Additional file 1.**

## Data Availability

The datasets used and/or analysed during the current study are available from the corresponding author on reasonable request.
