# Supplementary material for: Risk factors associated with physician trainee concern over missed educational opportunities during the COVID-19 pandemic
Source: BMC Med Educ. 2021 Apr 17;21:216. doi: 10.1186/s12909-021-02665-0 (PMC8052552; doi:10.1186/s12909-021-02665-0)
Supplement: Supplementary file 1 — Additional file 1. [file 12909_2021_2665_MOESM1_ESM.pdf]

Risk factors associated with physician trainee concern over missed educational opportunities during the COVID-19 pandemic

Sunny S. Lou, MD, PhD[1], Charles W. Goss, PhD[2], Bradley A. Evanoff, MD, MPH[3], Jennifer G. Duncan, MD[4], Thomas Kannampallil, PhD[1,5]

[1]Department of Anesthesiology, [2]Division of Biostatistics, [3]Department of Medicine. [4]Department of Pediatrics, [5]Institute for Informatics and Department of Anesthesiology

Washington University School of Medicine, St Louis, Missouri.

Corresponding Author:
Thomas Kannampallil, PhD
660 S. Euclid Avenue, Campus Box 8054
Washington University School of Medicine
St Louis, Missouri 63110
(314) 273-7801
thomas.k@wustl.edu

Resize font:

Assessing Physician Trainee Wellness during a Pandemic

The purpose of this survey to understand the impact of the COVID-19 pandemic on the well-being of physician trainees.

(Survey instance 2 of 3)

Please create your keyword using the following guidelines:

1. **First three letters of your mothers maiden name**
2. **First three letters of your city of birth**
3. **Your 2 digit birth DAY**

Keyword

Example:
"Smith"; "New Orleans"; "05/05/1980"
= "sminew05"

* must provide value

Are you a

- (•) Resident
- ( ) Fellow

reset

Please select your Residency program

- ( ) Anesthesiology
- ( ) Pathology
- ( ) Dermatology
- ( ) Emergency Medicine
- ( ) General Surgery
- ( ) Internal Medicine
- ( ) Medical Genetics
- ( ) Neurological Surgery
- ( ) Neurology
- ( ) Child Neurology
- ( ) Nuclear Medicine
- ( ) Obstetrics & Gynecology
- ( ) Ophthalmology
- ( ) Orthopedic Surgery
- ( ) Otolaryngology
- ( ) Pediatrics
- ( ) Physical Medicine & Rehab
- ( ) Plastic Surgery
- ( ) Psychiatry
- ( ) Radiation Oncology
- ( ) Radiology
- ( ) Urology

reset

Year in program:

Sex:

- Male
- Female
- Gender diverse
- Prefer not to say

reset

Race:

- White
- Black or African American
- Asian
- Native American or Alaskan
- Hispanic or Latino
- Other
- Prefer not to say

reset

Marital Status:

- Married
- Domestic partner
- Single
- Other

reset

Do you have children living at home with you?

- Yes (selected)
- No

reset

How many Children? 2

Child 1 Age Infant (0-3)

Child 1 Age Elementary school (6-10)

Do you have elderly parents or relatives that you are caring for?

- Yes (selected)
- No

reset

Are they local?

- Yes
- No (selected)

reset

Currently, how stressed are you about

	Not at all	A little	Somewhat	Quite a bit	Extremely
**Childcare?**	◯	◯	◯	◯	◯ reset
**Home schooling?**	◯	◯	◯	◯	◯ reset
**Care for your relatives?**	◯	◯	◯	◯	◯ reset
**Your personal finances?**	◯	◯	◯	◯	◯ reset
**Missed educational opportunities?**	◯	◯	◯	◯	◯ reset
**Your clinical competency in your specialty?**	◯	◯	◯	◯	◯ reset
**Your competency/knowledge about caring for COVID patients?**	◯	◯	◯	◯	◯ reset
**Job opportunities?**	◯	◯	◯	◯	◯ reset

The following questions are related to your work/life activities. Answer the following questions with respect to your work-life situations in the past 2 weeks:

How often do the demands of your job interfere with your family life?

◯ Often
◯ Sometimes
◯ Rarely
◯ Never

reset

How often do the demands of your family interfere with your work on the job?

◯ Often
◯ Sometimes
◯ Rarely
◯ Never

reset

How hard is it to take time off during your work to take care of personal or family matters?

◯ Not at all hard
◯ Not too hard
◯ Somewhat hard
◯ Very hard

reset

After an average workday, about how many hours do you have to relax or pursue activities that you enjoy?

Number of hours (round to nearest hour)

In the past 2 weeks, what best describes the duties where you spent the majority of your time? (check one)

- ◯ Education (at home)
- ◯ Clinical (inpatient)
- ◯ Clinical (outpatient)
- ◯ Clinical (ICU)
- ◯ Clinical (ER)
- ◯ Clinical (work from home)
- ◯ Clinical (hospital services with patient contact) (e.g., anesthesiology, surgery)
- ◯ Clinical (hospital services with no patient contact) (e.g., pathology, radiology)
- ◯ Research (on campus)
- ◯ Research (from home)
- ◯ Quarantined

reset

In your current clinical role, are you seeing or caring for patients that are being tested for Covid-19?

- ◯ Yes
- ◯ No

reset

In your current clinical role, are you seeing or caring for patients that are that are positive for Covid-19?

- ◯ Yes
- ◯ No

reset

How true do you feel the following statements are about you at work during the past 2 weeks

	Not true at all	Somewhat true	Moderately true	Very true	Completely true	
I feel happy at work	◯	◯	◯	◯	◯	reset
I feel worthwhile at work	◯	◯	◯	◯	◯	reset
My work is satisfying to me	◯	◯	◯	◯	◯	reset
I feel in control when dealing with difficult problems at work	◯	◯	◯	◯	◯	reset
My work is meaningful to me	◯	◯	◯	◯	◯	reset
I am contributing professionally (e.g., patient care, research, and leadership in ways I value the most)	◯	◯	◯	◯	◯	reset

To what degree have you experienced the following: during the past 2 weeks, my job has contributed to me feeling:

	Not at all	Very little	Moderately	A lot	Extremely	
Less empathetic with my patients	◯	◯	◯	◯	◯	reset
Less empathetic with my colleagues	◯	◯	◯	◯	◯	reset
Less sensitive to others' feelings/emotions	◯	◯	◯	◯	◯	reset

Less interested in talking with my patients	○	○	○	○	○	reset
Less connected with my patients	○	○	○	○	○	reset
Less connected with my colleagues	○	○	○	○	○	reset

To what degree have you experienced the following: during the past 2 weeks, I have felt:

	Not at all	Very little	Moderately	A lot	Extremely	
A sense of dread when I think about work I have to do	○	○	○	○	○	reset
Physically exhausted at work	○	○	○	○	○	reset
Lacking in enthusiasm at work	○	○	○	○	○	reset
Emotionally exhausted at work	○	○	○	○	○	reset

To what degree have you experienced the following: during the past 2 weeks:

	Did not apply to me at all	Applied to me to some degree, or some of the time	Applied to me to a considerable degree or a good part of time	Applied to me very much or most of the time	
I found it hard to wind down	○	○	○	○	reset
I was aware of dryness of my mouth	○	○	○	○	reset
I couldn't seem to experience any positive feeling at all	○	○	○	○	reset
I experienced breathing difficulty (e.g., excessively rapid breathing, breathlessness in the absence of physical exertion)	○	○	○	○	reset
I found it difficult to work up initiative to do things	○	○	○	○	reset
I tended to over-react to situations	○	○	○	○	reset
I experienced trembling (e.g., in the hands)	○	○	○	○	reset
I felt that I was using a lot of nervous energy	○	○	○	○	reset
I was worried about situations in which I might panic and make a fool of myself	○	○	○	○	reset
I felt I had nothing to look forward to	○	○	○	○	reset
I found myself getting agitated	○	○	○	○	reset
I found it difficult to relax	○	○	○	○	reset
I felt down-hearted and blue	○	○	○	○	reset
I was intolerant of anything that kept me from getting on with what I was doing	○	○	○	○	reset

I felt I was close to panic	◯	◯	◯	◯	reset
I was unable to become enthusiastic about anything	◯	◯	◯	◯	reset
I felt I wasn't worth much as a person	◯	◯	◯	◯	reset
I felt I was rather touchy	◯	◯	◯	◯	reset
I was aware of the action of my heart in the absence of physical exertion (e.g., a sense of heart rate increase, heart missing a beat)	◯	◯	◯	◯	reset
I felt scared without any good reason	◯	◯	◯	◯	reset
I felt that life was meaningless	◯	◯	◯	◯	reset

Please answer the following questions regarding your program:

	Strongly disagree	Disagree	Agree	Strongly Agree	
My program director is concerned about the welfare of people around him/her	◯	◯	◯	◯	reset
My program director pays attention to what I am saying	◯	◯	◯	◯	reset
My program director is helpful in getting the job done	◯	◯	◯	◯	reset
My program director is successful in getting people to work together	◯	◯	◯	◯	reset

Below is a list of difficulties people sometimes have after stressful life events. Please read each item, and then indicate how distressing each difficulty has been for you during the past seven days with respect to the current COVID-19 pandemic. How much have you been distressed by the current current COVID-19 pandemic?

	Not at all	A little bit	Moderately	Quite a bit	Extremely	
Any reminder brought back feelings about it	◯	◯	◯	◯	◯	reset
I had trouble staying asleep	◯	◯	◯	◯	◯	reset
Other things kept making me think about it.	◯	◯	◯	◯	◯	reset
I felt irritable and angry	◯	◯	◯	◯	◯	reset
I avoided letting myself get upset when I thought about it or was reminded of it	◯	◯	◯	◯	◯	reset
I thought about it when I didn't mean to	◯	◯	◯	◯	◯	reset
I felt as if it hadn't happened or wasn't real	◯	◯	◯	◯	◯	reset
I stayed away from reminders of it	◯	◯	◯	◯	◯	reset
Pictures about it popped into my mind	◯	◯	◯	◯	◯	reset

I was jumpy and easily startled	◯	◯	◯	◯	◯	reset
I tried not to think about it	◯	◯	◯	◯	◯	reset
I was aware that I still had a lot of feelings about it, but I didn't deal with them	◯	◯	◯	◯	◯	reset
My feelings about it were kind of numb	◯	◯	◯	◯	◯	reset
I found myself acting or feeling like I was back at that time	◯	◯	◯	◯	◯	reset
I had trouble falling asleep	◯	◯	◯	◯	◯	reset
I had waves of strong feelings about it	◯	◯	◯	◯	◯	reset
I tried to remove it from my memory	◯	◯	◯	◯	◯	reset
I had trouble concentrating	◯	◯	◯	◯	◯	reset
Reminders ofit caused me to have physical reactions, such as sweating, trouble breathing, nausea, or a pounding heart	◯	◯	◯	◯	◯	reset
I had dreams about it	◯	◯	◯	◯	◯	reset
I felt watchful and on-guard	◯	◯	◯	◯	◯	reset
I tried not to talk about it	◯	◯	◯	◯	◯	reset

Are you aware of or have you used one of the following services in the past 6 months?

	Used	Didn't Use	Didn't Use / Not Aware	
GME Wellness website	◯	◯	◯	reset
GME Wellness Counseling	◯	◯	◯	reset
Covid-19 Wellness Zoom support sessions	◯	◯	◯	reset
WashU Wellness Connection programs	◯	◯	◯	reset
BJC Total Rewards wellness options	◯	◯	◯	reset
Covid-19 emergency housing on campus	◯	◯	◯	reset
Emergency childcare services for COVID	◯	◯	◯	reset

Submit
